# Dietary Fatty Acids and Antinuclear Antibodies Among Adults with Arthritis in the United States: NHANES 1999–2004

**DOI:** 10.3390/nu17060934

**Published:** 2025-03-07

**Authors:** Jie Guo, Yifei Yu, Jiaqi Su, Fazheng Ren, Juan Chen

**Affiliations:** 1 Department of Nutrition and Health, Key Laboratory of Precision Nutrition and Food Quality, China Agricultural University, Beijing 100091, China; guojie@cau.edu.cn (J.G.);; 2 Department of Health Sciences and Technology, ETH Zurich, 8092 Zurich, Switzerland

**Keywords:** arthritis, dietary fatty acid, omega-3 PUFAs, antinuclear antibodies, NHANES

## Abstract

**Background**: This study investigated the link between daily fatty acid intake and antinuclear antibody (ANA) levels, a marker of immune dysregulation and autoimmune diseases, in individuals with self-reported arthritis. **Methods**: From the US National Health and Nutrition Examination Survey (NHANES) 1999–2004, 829 participants who self-reported arthritis and had autoantibody measurements were selected. Dietary fatty acids were collected via two 24 h dietary recall interviews conducted by trained interviewers. ANA levels were collected by measuring IgG autoantibodies targeting cellular antigens. **Results**: In a multi-adjusted logistic model, the odds ratio (OR) of the highest tertile of omega-3 intake (with omega-3 intake > 1.60 g/day) for the probability of ANA positivity was 0.43 (95% CI: 0.19–0.96) compared to those in the lowest tertile (≤0.92 g/day). However, we did not observe statistically significant results for other fatty acids. **Conclusions**: Our findings highlight the potential of dietary omega-3 PUFAs to modulate immune function and lower the risk of ANA positivity in individuals with arthritis.

## 1. Introduction

Immune-inflammatory diseases represent a diverse spectrum of disorders characterized by chronic inflammation, immune system dysregulation, and tissue damage. Among these, arthritis encompasses a group of debilitating conditions including rheumatoid arthritis and osteoarthritis, which significantly impact quality of life [1,2]. Arthritis manifests as persistent joint pain, swelling, and progressive cartilage destruction, ultimately resulting in reduced physical function and a substantial socioeconomic burden. The pathophysiology of arthritis arises from a complex interplay of genetic, environmental, and lifestyle factors. Notably, diet has emerged as a modifiable factor influencing disease progression and symptom management [3].

Dietary fatty acids, particularly polyunsaturated fatty acids (PUFAs), have garnered considerable attention for their immunomodulatory and anti-inflammatory effects [4,5,6]. Previous research has highlighted the role of omega-3 PUFAs, such as eicosapentaenoic acid (EPA) and docosahexaenoic acid (DHA), in mitigating inflammatory responses by modulating cytokine production, reducing oxidative stress, and promoting the resolution of inflammation [7,8,9]. Conversely, omega-6 fatty acids, while generally associated with pro-inflammatory effects, may exhibit more nuanced effects depending on their balance with omega-3 fatty acids [10,11]. Observational and interventional studies have consistently reported the association between increased dietary intake or supplementation of specific fatty acids and improvements in arthritis symptoms, including a reduction in joint pain, stiffness, and systemic inflammation [12]. However, despite these promising findings, the impact of broader fatty acid profiles on arthritis, particularly their interplay with immune markers, remains insufficiently underexplored.

Antinuclear antibodies (ANAs) are autoantibodies targeting nuclear components of cells and serve as hallmark biomarkers for systemic autoimmune diseases, including systemic lupus erythematosus and rheumatoid arthritis [13,14,15]. The presence of ANAs indicates dysregulation of immune tolerance and heightened autoimmune activity, making them critical diagnostic and prognostic indicators for arthritis-related conditions [16]. Beyond their diagnostic role, ANAs may contribute to disease pathogenesis by forming immune complexes that exacerbate inflammation and tissue damage [17,18]. Their prevalence varies among populations, with significant implications for identifying individuals at risk of developing arthritis or monitoring disease activity. Understanding the factors that influence ANA levels, including dietary components, is therefore crucial for elucidating the complex interactions between nutrition, immunity, and arthritis.

We hypothesized that it is possible for ANA levels to correlate with different types of fatty acids in the population. This study investigates the link between daily intake of 19 fatty acids and ANA presence in US adults with arthritis, using NHANES 1999–2004 data. It aims to provide insights into dietary strategies for modulating immune responses and slowing arthritis progression.

## 2. Materials and Methods

The National Health and Nutrition Examination Survey, a program by the Centers for Disease Control and Prevention in collaboration with the National Center for Health Statistics, assesses the health and nutrition of the U.S. population living outside of institutions. Using a stratified multistage probability sampling method, NHANES collects comprehensive data through household interviews, physical examinations at mobile examination centers, and laboratory analyses of biological specimens. To ensure adequate representation of key demographic subgroups, such as racial and ethnic minorities, children, and older adults, the survey incorporates an oversampling strategy. NHANES operates as a continuous program, with data released in biennial cycles. Study protocols are reviewed and approved by an Institutional Review Board, with informed consent obtained from all participants [19].

### 2.1. Study Population

**Figure 1:**
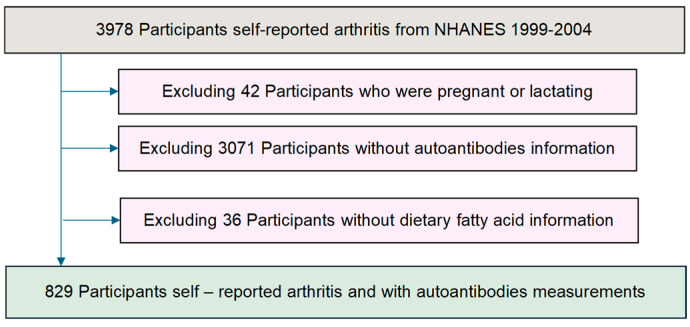
Flow chart of the study population.

The study utilized data collected from the NHANES 1999–2004, focusing on individuals who self-reported arthritis. Initially, a cohort of 3978 participants with self-reported arthritis was identified. To minimize potential confounding, we further excluded pregnant and lactating women (n = 42), participants without autoantibody measurements (n = 3071), and participants without dietary fatty acid intake information (n = 36). After these exclusions, the final analytical cohort comprised 829 participants with self-reported arthritis, and complete autoantibody measurements and dietary fatty acid intake data were included in the analysis (Figure 1).

### 2.2. Assessment of Dietary Fatty Acids

Dietary fatty acids were estimated using data from one (1999–2000 and 2001–2002) or two (2003–2004) 24 h dietary recall interviews. Since cycle 2003–2004, the dietary recall interviews have been conducted twice for each participant: The first interview was conducted at the Mobile Examination Center, followed by a second interview via phone, 3 to 10 days later. Nutrient and micronutrient intake were assessed using data from the U.S. Department of Agriculture database, while saturated fatty acid (SFA), monounsaturated fatty acid (MUFA), polyunsaturated fatty acid (PUFA), and decanoic acid were derived from the NHANES 1999–2004 database directly. Moreover, omega-3 PUFA (ω-3 PUFA) was calculated by summing the intake of alpha-linolenic acid, stearic acid, eicosapentaenoic acid, docosapentenoic acid, and docosahexaenoic acid. Omega-6 PUFA (ω-6 PUFA) was calculated by summing the intake of linoleic acid and arachidonic acid. The decanoic acid to ω-3 PUFA ratio and ω-3 to ω-6 ratio were calculated. To facilitate analysis, the dietary intakes of these fatty acids, and the ratios between them, were then categorized into tertiles for further analysis [20,21,22].

### 2.3. Assessment of ANA Test

ANA titers were determined using stored serum samples from a one-third subsample of NHANES participants aged 12 years and older. ANA screening was conducted using the indirect immunofluorescence assay on HEp-2 cells [23]. IgG autoantibodies targeting cellular antigens were detected, and fluorescence staining intensity was graded on a scale from 0 to 4. Samples with a staining intensity ≥ 3 are defined as ANA positive, and samples with a staining intensity of less than 3 are ANA negative. The cellular staining patterns, such as speckled, nucleolar, and homogeneous, were assessed according to their anatomical distribution of intracellular antigens and recorded using a reference gallery. This approach, combining immunofluorescence and immunoprecipitation, ensured precise identification of ANA titers and specificities, providing a reliable estimate of autoantibody prevalence and characteristics within the U.S. population during the study period [24,25].

### 2.4. Covariates

To account for potential confounding factors, relevant demographic and socioeconomic information was obtained from the NHANES study to account for potential covariates. In the multi-adjusted model, we included age, sex, race/ethnicity (non-Hispanic White, non-Hispanic Black, Mexican American, and other Hispanic or other race), highest education level (less than high school, high school or equivalent, college or above), smoking status (current, former, never), alcohol consumption status (non-drinker, drinker), marital status (married or living with a partner, not married nor living with a partner), and poverty-to-income ratio (categorized as <1.3, 1.3–3.5, and ≥3.5). These variables were selected due to their potential confounding effect for the association between fatty acids and ANA level [26,27].

### 2.5. Statistical Analysis

The 6-year survey weights extracted from the ANA database were systematically applied across all analyses to appropriately adjust for NHANES’s complex sampling design. Baseline characteristics were described as weighted means and standard errors or percentages among total, ANA-negative, and ANA-positive participants.

Logistic regression models were applied to examine the odds ratio (OR) and 95% confidence interval (CI) for the relationship between ANA status and dietary fatty acids, categorized into tertiles, with the lowest tertile serving as the reference group. In the logistic regression models, we first controlled for age, sex, and race/ethnicity (Model 1), then made further adjustments for education level, marital status, and poverty-to-income ratio (Model 2), and subsequently adjusted for drinking status, smoking status, and total energy intake (Model 3). We further explored the ORs of continuous dietary fatty acid with ANA status using the restricted cubic spline with four knots at the 5th, 35th, 65th, and 95th percentiles.

Statistical analyses were conducted with SAS 9.4, employing two-tailed tests with a significance threshold of *p* < 0.05. Weighted estimates, including proportions and means (±SE), were generated through the SURVEYFREQ and SURVEYMEANS procedures. Multiple comparison adjustments were implemented using the Bonferroni method. The SURVEYLOGISTIC procedure was used to calculate the OR (95% CI). Data were analyzed from November 2024 to January 2025.

### 2.6. Ethical Approval and Consent to Participate

All analyses were conducted using publicly available databases. The Institutional Review Board for the National Center for Health Statistics approved the NHANES, which was performed in accordance with the Declaration of Helsinki. All participants provided written informed consent before data collection.

## 3. Results

### 3.1. Baseline Characteristics

Table 1 presents the baseline characteristics of the study population stratified by ANA status. The mean age of participants was 59.8 (standard deviation: 0.6) years and 60.9% were females. Participants who were ANA positive were more likely to be females, to never have been smokers, and to have lower levels of omega 3 PUFAs, omega 6 PUFAs, MUFAs, and PUFAs.

### 3.2. Associations of Dietary Fatty Acids Intake with the Probability of Being ANA Positive

Table 2 presents the ORs and 95% CIs for the association between various dietary fatty acids and ANA positivity. In Model 1, the OR (95% CI) of the highest tertile of omega-3 intake (with omega-3 intake > 1.60 g/day) for the probability of being ANA positive was 0.62 (95% CI: 0.40–0.96) compared to those in the lowest tertile (≤0.92 g/day), which was still statistically significant after multi-adjustment (0.43 [95% CI: 0.19–0.96]). However, other fatty acids such as decanoic acid, omega-6 PUFAs, SFAs, MUFAs, and total PUFAs did not demonstrate any significant associations with ANA positivity.

**Figure 2:**
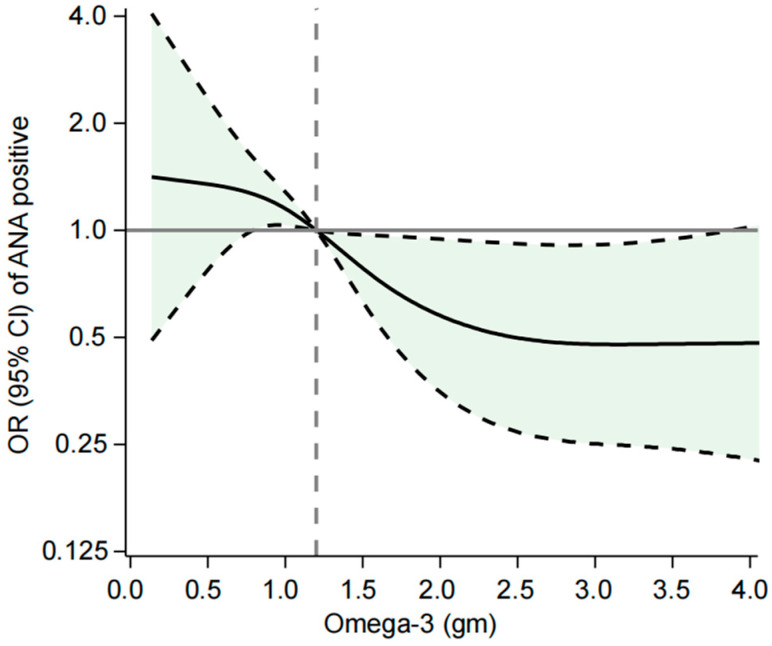
OR (95% CI) of ANA positivity in relation to dietary omega-3 intake.

The associations between dietary intake of 19 fatty acids and the probability of ANA positivity were explored, and the results reveal that only omega-3 polyunsaturated fatty acids have a significant protective effect against ANA positivity. Individuals in the highest tertile of omega-3 intake (>1.60 g/day) exhibited a substantially reduced likelihood of ANA positivity compared to those in the lowest tertile (≤0.92 g/day). In Model 3, the OR for ANA positivity in the highest tertile was 0.43 (95% CI: 0.19–0.96), suggesting that higher omega-3 consumption is associated with a significantly lower risk of ANA positivity. These findings highlight the unique and beneficial role of omega-3 fatty acids in reducing the risk of ANA positivity, potentially through their anti-inflammatory and immunomodulatory properties. However, other dietary fatty acids appear to have no significant impact on ANA status (Figure 2).

The odds ratio (OR) and 95% confidence interval (CI) were calculated using multivariable logistic regression models with restricted cubic splines. The model was adjusted for age, sex, race/ethnicity, education level, marital status, poverty-to-income ratio, drinking status, smoking status, and total energy intake. The *p*-value for overall association and *p*-value for linear association were both <0.05.

## 4. Discussion

Our study explored the relationships between ANA positivity and 19 dietary fatty acids, including omega-3 and omega-6 polyunsaturated fatty acids, as well as monounsaturated and saturated fatty acids. The most significant finding was a strong inverse association between dietary omega-3 PUFA intake and the probability of ANA positivity. Participants in the highest tertile of omega-3 intake (>1.60 g/day) demonstrated a 57% reduced likelihood of ANA positivity compared to those in the lowest tertile (≤0.92 g/day), with the relationship remaining robust across multiple adjustment models. In contrast, none of the other dietary fatty acids, including omega-6 PUFAs, SFAs, MUFAs, or their ratios, exhibited statistically significant associations with ANA positivity. These findings underscore the role of omega-3 PUFAs in modulating immune function and potentially mitigating autoimmune risk.

The inverse association between omega-3 intake and ANA positivity aligns with prior research demonstrating the anti-inflammatory and immunomodulatory effects of omega-3 PUFAs. For example, research consistently shows that omega-3 fatty acids, especially EPA and DHA, are linked to lower levels of pro-inflammatory cytokines like TNF-α, IL-1β, and IL-6 [28,29,30]. These cytokines play critical roles in the pathogenesis of autoimmune diseases. Furthermore, omega-3 fatty acid supplementation has demonstrated reductions in disease activity and symptom severity in autoimmune diseases such as rheumatoid arthritis [31], lupus [32], and multiple sclerosis [33]. While direct studies linking omega-3 PUFA intake to ANA positivity are limited, the observed protective effects are consistent with their role in immune regulation. Notably, this study did not find significant associations between omega-6 PUFAs and ANA positivity. This result is consistent with mixed evidence regarding omega-6 fatty acids and inflammation [34]. Omega-6 PUFAs have a complex relationship with inflammation. While they are precursors to pro-inflammatory eicosanoids (e.g., prostaglandins and leukotrienes), they also contribute to the generation of anti-inflammatory mediators under certain conditions [35,36]. The balance between omega-6 and omega-3 fatty acids intake is crucial for maintaining immune homeostasis, and prior studies have suggested that an increased ratio of omega-6 to omega-3 intake may exacerbate inflammatory processes [35,37]. However, no significant effects of this ratio on ANA positivity were observed, potentially due to the relatively low omega-6 intake in the study population or unaccounted for confounders. Current direct studies directly examining the relationship between dietary fatty acids and ANA positivity remain limited. However the prevalence of ANA has been explored in the context of other inflammatory and autoimmune diseases. For example, in Hispanic children with NAFLD, ANA positivity was associated with insulin resistance and lower levels of HDL cholesterol levels [38]. Additionally, serum linoleic acid concentrations were found to correlate positively with ANA titers in SLE patients [39].

The observed protective effect of omega-3 PUFAs on ANA positivity may be mediated by several interconnected mechanisms. Based on previous literature studies, omega-3 PUFAs are well-documented modulators of inflammatory pathways. They are integrated into cell membranes, where they compete with omega-6 PUFAs for cyclooxygenase and lipoxygenase enzyme substrates. This leads to a shift in eicosanoid profiles, favoring less pro-inflammatory and more anti-inflammatory eicosanoids [40,41]. This shift in eicosanoid profiles may reduce systemic inflammation and autoantibody production, both of which are critical contributors to ANA positivity and the pathogenesis of autoimmune diseases. Beyond these, omega-3 PUFAs may further reduce ANA levels by altering lipid raft dynamics in immune cell membranes. Importantly, omega-3 PUFAs are associated with downregulation of the nuclear factor κB (NFκB) signaling pathway, which is a master switch regulating the expression of many pro-inflammatory genes. Omega-3 PUFAs inhibit NF-κB/MAPK signaling, thereby inhibiting autoreactive B cell activation and nuclear antigen exposure [42,43]. Omega-3 PUFAs also modulate immune cell function. They can reduce the activation of pro-inflammatory M1 macrophages and enhance the differentiation of anti-inflammatory M2 macrophages [44,45,46]. Similarly, omega-3 PUFAs inhibit the activation of Th17 cells, a subset of the T-helpers cell subset implicated in autoimmunity, while enhancing the activity of regulatory T cells, which support immune tolerance [44]. These immunomodulatory effects could directly impact ANA production and the development of autoimmune conditions. Furthermore, omega-3 PUFAs may exert their effects through epigenetic modifications [47]. Emerging evidence suggests that omega-3 fatty acids can alter DNA methylation, histone acetylation, and microRNA expression, leading to changes in gene expression patterns involved in inflammation and immunity [48,49]. These epigenetic effects could provide a mechanistic link between dietary omega-3 intake and reduced ANA positivity. Metabolically, EPA acts as an endogenous PPARγ ligand to reprogram dendritic cell metabolism toward fatty acid oxidation, inhibiting mTORC1-mediated cytokine release and enhancing Nrf2 activity to reduce ROS-induced DNA damage and neutrophil extracellular trap formation, thereby limiting modified nuclear antigen generation [50]. Moreover, the gut microbiota may serve as a mediator of the protective effects of omega-3 fatty acids. Omega-3 PUFAs are known to alter gut microbiota composition, enhancing the abundance of beneficial bacteria like Bifidobacterium and Lactobacillus while decreasing pro-inflammatory taxa [51,52]. Healthy gut microbiota can strengthen the intestinal barrier, reduce systemic inflammation, and enhance immune regulation, all of which may contribute to lower ANA positivity.

A strength of this study is that we used a nationally representative sample of the U.S. population for the analyses, with various dietary fatty acids and ANA assessed following standardized procedures. Nevertheless, some limitations need to be acknowledged. First, dietary data were collected based on self-reported 24 h dietary recalls, which are subject to recall bias and may not accurately represent habitual intake. Considering that people tend to think in a positive way, they will overestimate their fatty acid intake and may be wrongly classified, which will lead to an underestimation of our results. The reliance on a single or limited number of dietary recalls may also fail to capture long-term dietary patterns. Second, the form of consumption of unsaturated fatty acids and the duration of consumption may also matter. However, we could not consider these factors because they were not collected in the NHANES. Further studies with more detailed information are warranted to comprehensively assess the association between fatty acids and ANA levels. Third, we could not conclude the causal association due to the cross-sectional study design. Further studies using longitudinal data are needed to confirm these findings and elucidate causality. Then, the sample size of ANA-positive individuals was relatively small, which may limit the generalizability of our results. To validate our findings and investigate potential interactions between dietary fatty acids and other demographic or lifestyle factors, larger studies with more diverse populations are needed. Finally, the data used in this study were derived from the NHANES 1999–2004 cycles, and dietary patterns may have shifted since then. Future studies should examine more recent datasets to determine whether these associations persist in contemporary populations.

## 5. Conclusions

This study provides novel evidence associating higher dietary omega-3 PUFA intake with a reduced likelihood of ANA positivity in individuals with arthritis. These finding suggesting a potential role for omega-3 fatty acids in modulating immune function and lowering autoimmune risk. These results highlight the importance of dietary interventions as a strategy for preventing and managing immune-related conditions. In addition, further studies, such as animal experiments and randomized clinical trials, are warranted to elaborate the underlying mechanisms of the protective effect of omega-3 PUFAs on autoimmune diseases and to explore the therapeutic effect of omega-3 PUFAs on autoimmune diseases in the population. Further research is warranted to confirm these results, elucidate the underlying biological mechanisms, and examine the broader implications of omega-3 fatty acid intake in the context of autoimmune disease contexts.

## Figures and Tables

**Table 1:** Baseline characteristics of study population by ANA status (N = 865).

Characteristics	Total	ANA Positive	ANA Negative	*p*-Value
**Age, mean (SD), years**	59.8 (0.6)	62.4 (1.5)	59.3 (0.6)	0.099
**Sex (%)**				0.018
Female	492 (60.9)	88 (70.1)	404 (59.2)	
Male	337 (39.1)	46 (29.9)	291 (40.8)	
**Race/Ethnicity (%)**				0.530
Mexican American	142 (3.6)	19 (3.3)	123 (3.6)	
Non-Hispanic White	490 (77.1)	76 (73.6)	414 (77.8)	
Non-Hispanic Black	147 (11.4)	29 (14.9)	118 (10.7)	
Other Hispanic or other race	50 (7.9)	10 (8.2)	40 (7.9)	
**Married status (%)**				0.178
Married or living with a partner	472 (63.2)	68 (56.6)	404 (64.4)	
Not married nor living with a partner	332 (36.8)	63 (43.4)	269 (35.6)	
**Poverty-to-income ratio (%)**				0.485
<1.3	240 (24.2)	34 (20.2)	206 (24.9)	
1.3–3.5	313 (40.2)	54 (45.8)	259 (39.2)	
≥3.5	211 (35.6)	33 (34.0)	178 (35.9)	
**Highest education (%)**				0.859
Less than high school	319 (27.2)	49 (26.8)	270 (27.3)	
High school or equivalent	193 (27.5)	33 (29.6)	160 (27.1)	
College or above	315 (45.3)	52 (43.7)	263 (45.6)	
**Smoking status (%)**				<0.001
Current	162 (20.8)	17 (11.6)	145 (22.5)	
Former	302 (36.3)	45 (30.4)	257 (37.4)	
Never	365 (42.9)	72 (58.0)	293 (40.1)	
**Drinking status (%)**				0.242
Non-drinker	296 (34.7)	41 (28.9)	255 (35.7)	
Drinker	505 (65.3)	85 (71.1)	420 (64.3)	
**Total energy, mean (SD), kcal**	1987 (42.5)	1874.9 (78.5)	2008.9 (48.6)	0.182
**Omega 3 PUFAs, mean (SD), g**	1.6 (0.0)	1.4 (0.1)	1.6 (0.1)	0.031
**Decanoic (g)**	0.4 (0.0)	0.4 (0.0)	0.4 (0.0)	0.620
**Decanoic: omega 3 PUFAs ratio**	0.3 (0.0)	0.3 (0.0)	0.3 (0.0)	0.501
**Omega 6 PUFAs (g)**	14.4 (0.4)	12.7 (0.6)	14.7 (0.4)	0.014
**Omega 3: omega 6 PUFAs ratio**	0.1 (0.0)	0.1 (0.0)	0.1 (0.0)	0.021
**SFAs (g)**	25.1 (0.7)	23.2 (1.3)	25.5 (0.8)	0.170
**MUFAs (g)**	28.5 (0.8)	25.7 (1.3)	29.1 (0.9)	0.045
**PUFAs (g)**	16.2 (0.4)	14.3 (0.7)	16.5 (0.5)	0.016

Abbreviations: ANA, antinuclear antibody; SFA, saturated fatty acid; MUFA, monounsaturated fatty acid; PUFA, polyunsaturated fatty acid. Data are presented as weighted means (standard error) for continuous variables and weighted percentages for categorical variables.

**Table 2:** Odds ratios and 95% confidence intervals of dietary fatty acid intake for ANA positivity.

Dietary Fatty Acid	Model 1	Model 2	Model 3
**Decanoic (g)**			
Tertile 1 (≤0.17)	Reference	Reference	Reference
Tertile 2 (>0.17, ≤0.42)	0.66 (0.36–1.21)	0.59 (0.31–1.16)	0.58 (0.28–1.21)
Tertile 3 (>0.42)	0.99 (0.54–1.83)	0.98 (0.51–1.86)	0.97 (0.45–2.08)
**Omega-3 (g)**			
Tertile 1 (≤0.92)	Reference	Reference	Reference
Tertile 2 (>0.92, ≤1.60)	0.67 (0.41–1.11)	0.65 (0.38–1.13)	0.59 (0.30–1.17)
Tertile 3 (>1.60)	0.62 (0.40–0.96)	0.55 (0.35–0.86)	0.43 (0.19–0.96)
**Decanoic: omega 3 PUFAs ratio**			
Tertile 1 (≤0.14)	Reference	Reference	Reference
Tertile 2 (>0.14, ≤0.33)	1.20 (0.74–1.95)	1.16 (0.73–1.84)	1.14 (0.73–1.78)
Tertile 3 (>0.33)	1.57 (0.78–3.17)	1.70 (0.81–3.58)	1.64 (0.75–3.60)
**Omega-6 (g)**			
Tertile 1 (≤8.79)	Reference	Reference	Reference
Tertile 2 (>8.79, ≤15.13)	1.03 (0.67–1.59)	1.01 (0.63–1.64)	1.04 (0.57–1.89)
Tertile 3 (>15.13)	0.87 (0.52–1.45)	0.89 (0.51–1.53)	0.82 (0.36–1.85)
**Omega 3: omega 6 PUFAs ratio**			
Tertile 1 (≤0.09)	Reference	Reference	Reference
Tertile 2 (>0.09, ≤0.12)	1.21 (0.76–1.94)	1.11 (0.70–1.74)	1.22 (0.75–1.98)
Tertile 3 (>0.12)	0.78 (0.49–1.25)	0.66 (0.43–1.03)	0.71 (0.45–1.13)
**SFAs (g)**			
Tertile 1 (≤15.75)	Reference	Reference	Reference
Tertile 2 (>15.75, ≤25.41)	0.98 (0.60–1.62)	0.92 (0.57–1.49)	0.97 (0.53–1.78)
Tertile 3 (>25.41)	0.98 (0.55–1.75)	0.97 (0.55–1.72)	1.07 (0.41–2.75)
**MUFAs (g)**			
Tertile 1 (≤17.89)	Reference	Reference	Reference
Tertile 2 (>17.89, ≤29.67)	1.14 (0.68–1.91)	1.31 (0.82–2.09)	1.38 (0.81–2.35)
Tertile 3 (>29.67)	0.86 (0.48–1.56)	0.90 (0.49–1.67)	0.93 (0.40–2.20)
**PUFAs (g)**			
Tertile 1 (≤9.90)	Reference	Reference	Reference
Tertile 2 (>9.90, ≤17.15)	1.01 (0.64–1.59)	1.00 (0.60–1.67)	1.01 (0.53–1.91)
Tertile 3 (>17.15)	0.85 (0.52–1.41)	0.86 (0.51–1.45)	0.75 (0.34–1.68)

Abbreviations: ANA, antinuclear antibody; SFA, saturated fatty acid; MUFA, monounsaturated fatty acid; PUFA, polyunsaturated fatty acid. Model 1 adjusted for age, sex, and race/ethnicity. Model 2 further adjusted for education level, marital status, and poverty-to-income ratio. Model 3 further adjusted for drinking status, smoking status, and total energy intake.

## Data Availability

All relevant data in this study are publicly available on the National Health and Nutrition Examination Survey Homepage (https://www.cdc.gov/nchs/nhanes/index.html (accessed on 4 March 2025)). The corresponding author Dr Juan Chen (email address: chenjuan@cau.edu.cn) should be contacted for any requests (e.g., data used for all analyses; analytic code; any other materials used in the current study).

## Abbreviations

The following abbreviations are used in this manuscript:

ANA	antinuclear antibodies
CI	confidence interval
DHA	docosahexaenoic acid
EPA	eicosapentaenoic acid
MUFA	monounsaturated fatty acid
NHANES	National Health and Nutrition Examination Survey
OR	odds ratio
PUFA	polyunsaturated fatty acid
SFA	saturated fatty acid
